# Chikungunya Virus Vaccine Candidates with Decreased Mutational Robustness Are Attenuated *In Vivo* and Have Compromised Transmissibility

**DOI:** 10.1128/JVI.00775-19

**Published:** 2019-08-28

**Authors:** Lucía Carrau, Veronica V. Rezelj, María G. Noval, Laura I. Levi, Daniela Megrian, Herve Blanc, James Weger-Lucarelli, Gonzalo Moratorio, Kenneth A. Stapleford, Marco Vignuzzi

**Affiliations:** a Viral Populations and Pathogenesis Unit, Department of Virology, Institut Pasteur, CNRS UMR 3569, Paris, France; b Evolutionary Biology of the Microbial Cell Unit, Department of Microbiology, Institut Pasteur, Paris, France; c Department of Biomedical Sciences and Pathobiology, Virginia Polytechnic Institute and State University, Blacksburg, Virginia, USA; d Laboratorio de Inmunovirología, Institut Pasteur de Montevideo, Montevideo, Uruguay; e Laboratorio de Virología Molecular, Centro de Investigaciones Nucleares, Facultad de Ciencias, Universidad de la República, Montevideo, Uruguay; f Department of Microbiology, New York University School of Medicine, New York, New York, USA; University of Pittsburgh School of Medicine

**Keywords:** chikungunya, mutational robustness, vaccines

## Abstract

Chikungunya virus (CHIKV) is a reemerged arbovirus, a member of the *Togaviridae* family. It circulates through mosquito vectors mainly of the *Aedes* family and a mammalian host. CHIKV causes chikungunya fever, a mild to severe disease characterized by arthralgia, with some fatal outcomes described. In the past years, several outbreaks mainly caused by enhanced adaptation of the virus to the vector and ineffective control of the contacts between infected mosquito populations and the human host have been reported. Vaccines represent the best solution for the control of insect-borne viruses, including CHIKV, but are often unavailable. We designed live attenuated CHIKVs by applying a rational genomic design based on multiple replacements of synonymous codons. In doing so, the virus mutational robustness (capacity to maintain phenotype despite introduction of mutations to genotype) is decreased, driving the viral population toward deleterious evolutionary trajectories. When the candidate viruses were tested in the insect and mammalian hosts, we observed overall strong attenuation in both and greatly diminished signs of disease. Moreover, we found that the vaccine candidates elicited protective immunity related to the production of neutralizing antibodies after a single dose. During an experimental transmission cycle between mosquitoes and naive mice, vaccine candidates could be transmitted by mosquito bite, leading to asymptomatic infection in mice with compromised dissemination. Using deep-sequencing technology, we observed an increase in detrimental (stop) codons, which confirmed the effectiveness of this genomic design. Because the approach involves hundreds of synonymous modifications to the genome, the reversion risk is significantly reduced, rendering the viruses promising vaccine candidates.

**IMPORTANCE** Chikungunya fever is a debilitating disease that causes severe pain to the joints, which can compromise the patient’s lifestyle for several months and even in some grave cases lead to death. The etiological agent is chikungunya virus, an alphavirus transmitted by mosquito bite. Currently, there are no approved vaccines or treatments against the disease. In our research, we developed novel live attenuated vaccine candidates against chikungunya virus by applying an innovative genomic design. When tested in the insect and mammalian host, the vaccine candidates did not cause disease, elicited strong protection against further infection, and had low risk of reversion to pathogenic phenotypes.

## Abstract (precis)

Chikungunya fever is a debilitating disease that causes severe pain to the joints, which can compromise the patient’s lifestyle for several months and even in some grave cases lead to death. The etiological agent is chikungunya virus, an alphavirus transmitted by mosquito bite. Currently, there are no approved vaccines or treatments against the disease. In our research, we developed novel live attenuated vaccine candidates against chikungunya virus by applying an innovative genomic design. When tested in the insect and mammalian host, the vaccine candidates did not cause disease, elicited strong protection against further infection, and had low risk of reversion to pathogenic phenotypes.

## INTRODUCTION

Chikungunya virus (CHIKV) is a positive-strand RNA virus, a member of the *Alphavirus* genus, *Togaviridae* family. CHIKV is an arbovirus transmitted by mosquitoes (mainly the *Aedes* family) (1) to mammalian hosts through chains that involve both sylvatic and urban cycles. The virus was first identified in Tanzania (2, 3) though evidence points to multiple outbreaks where CHIKV was confused with dengue virus prior to the 1950s (4). Since its reemergence in the Indian Ocean islands/Asia and in the Americas in 2004/2005 and in 2013, respectively, CHIKV has expanded dramatically, reaching 111 countries on 5 continents, where autochthonous transmission was recorded (5). The disease affects millions of people, who may be asymptomatic or have symptoms ranging from mild to severe, including myalgia, arthralgia, fever, and rash, among others. CHIKV fever can also develop into a chronic disease, characterized by strong polyarthralgia, severely compromising a patient’s lifestyle (6). Fatal outcomes, while rare, have also been documented (7). Since the 1960s several efforts have been made to develop antiviral treatments and vaccines against CHIKV, and while some vaccine candidates are currently under clinical trials, no approved treatments or vaccines are yet available (8).

Live attenuated vaccines (LAVs) have been successfully used throughout the 20th century, preventing severe diseases and protecting millions of people. Even though their formulation is based on attenuated live viruses that can pose an intrinsic risk (like the LAV CHIKV candidate 181/25 which caused disease in vaccinees in clinical trials [9]), LAVs have proven to be better at eliciting long-lasting protection through effective humoral and cellular responses and require fewer doses (10, 11). In recent years, innovative research has focused on developing or improving LAVs by rationally attenuating viruses in order to reduce the reversion potential to minimal levels (12). A variety of approaches involve different ways in which the composition or positions of synonymous codons are modified in the viral genome. These strategies result in alterations in codon bias (13, 14), codon pair bias (15), dinucleotide composition (CpG and UpA) (16, 17), and mutational robustness (18, 19), all of which lead to attenuated viral phenotypes in several viral families (20). Our group has focused on mutational robustness, defined as the extent to which phenotype remains constant despite mutations in genotype (21). To decrease mutational robustness, we previously replaced leucine and serine codons in the genome of coxsackie virus B3 and influenza A virus with synonymous ones that are mutationally closer to become nonsense mutations (1 mutation away from stop, or “1-to-stop”). In this way, we generated viruses that were less tolerant to mutations, as evidenced by an increase in stop mutations in progeny viruses, which led to an attenuated phenotype (18).

Based on these results, we implemented this approach in CHIKV, with the ultimate goal of generating attenuated viruses that could be proposed as vaccine candidates. We thus replaced synonymous leucine and serine codons with the 1-to-stop codons in the structural open reading frame (ORF) of the CHIKV genome. To further increase the attenuation phenotype, we included 1-to-stop codons for arginine and glycine. Our recoded viruses showed strong attenuation in both insect and mammalian hosts while eliciting a protective immune response.

## RESULTS

### Rational design of CHIKV mutants and evaluation of replication kinetics.

**FIG 1:**
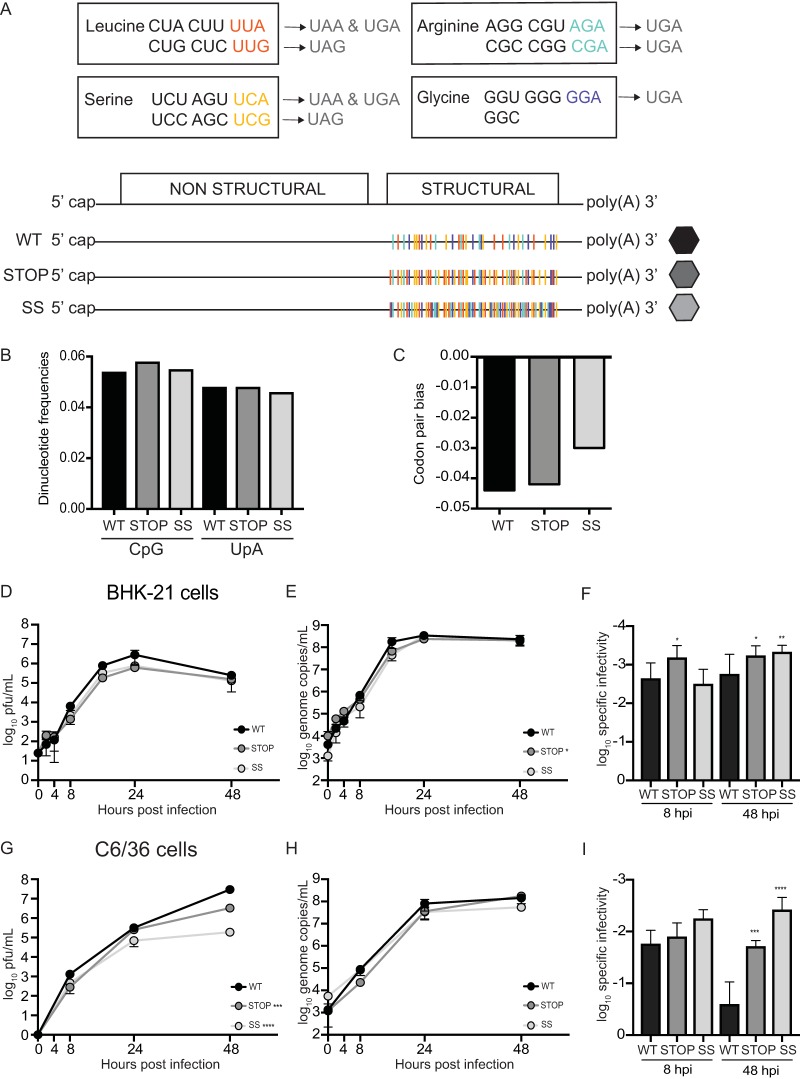
Design and production of Stop and SuperStop viruses and evaluation of replication kinetics. (A) Representation of the rationale and genomic design of our constructs. Synonymous codons in the 1-to-stop category for leucine (red), serine (yellow), arginine (green), and glycine (blue) are shown, followed by the stop codon they can reach after a single point mutation. Below, the chikungunya virus genome organization is shown, followed by a representation of wild-type (WT), Stop, and SuperStop (SS) viruses, where 1-to-stop synonymous codons in the structural open reading frame are depicted in color. (B) CpG and UpA dinucleotide frequencies for WT, Stop, and SS viruses. (C) Codon pair bias for WT, Stop, and SS viruses. (D and G) Replication kinetics of WT, Stop, and SS viruses at an MOI of 0.1 PFU/cell in BHK-21 cells and C6/36 cells. ***, *P* = 0.0005; ****, *P* < 0.0001. (E and H) Genome copy numbers of WT, Stop, and SS viruses during replication at different time points in BHK-21 cells (E) and C6/36 cells (H). Stop *, *P* = 0.022. For panels D, E, G, and H, mean values with standard errors of the means are shown (*n* = 3). Significance was determined by two-way analysis of variance with Tukey’s multiple-comparison test. (F and I) Specific infectivity of WT, Stop, and SS viruses in BHK-21 cells (F) and C6/36 cells (I). Mean values with standard deviation are shown (*n* = 3). Statistical significance is indicated as follows: 8 hpi *, *P* = 0.0110; 48 hpi *, *P* = 0.0180; 48 hpi **, *P* = 0.0052 (panel F); ***, *P* = 0.0010; ****, *P* < 0.0001 (panel I) (by one-way analysis of variance with Tukey’s multiple-comparison test). No statistical significance is indicated if *P* is >0.05.

Based on the results previously obtained by our group (18), we sought to generate CHIKV constructs that would have reduced mutational robustness, making them less able to tolerate the effects of mutations. If so, these viruses would bear an attenuated phenotype and therefore could represent good vaccine candidates. We followed a similar genomic design, in which we swapped synonymous codons to have evolutionary trajectories that would drive them toward stop mutations. In other words, we favored synonymous codons that have the highest likelihood of becoming a stop codon after the introduction of single nucleotide substitutions (1-to-stop group, 1 mutation away from stop). Having defined our 1-to-stop category of synonymous codons for serine (Ser), leucine (Leu), arginine (Arg), or glycine (Gly), we *de novo* synthesized CHIKV constructs bearing 1-to-stop codons in place of all other synonymous codons for the aforementioned amino acids in the structural protein-coding region of the genome. It is worth mentioning that, given that the structural ORF of CHIKV is translated into a polyprotein precursor (6), an introduction of a stop codon would render the ORF nonfunctional. Our first construct, named Stop virus, had all 1-to-stop synonymous codons (151 codons) for Leu and Ser in the structural region of the CHIKV genome (Fig. 1A). Our second construct, encoding SuperStop (SS) virus, had all 1-to-stop synonymous codons (285 codons) for leucine, serine, arginine, and glycine in the structural region of the CHIKV genome (Fig. 1A). Both constructs were designed in such a way that codon pair bias and CpG/UpA dinucleotide frequencies remained as close as possible to those of wild-type (WT) virus (Fig. 1B and C), such that the effects observed could be attributed to a greater extent to our genomic design rather than to other factors, as previously described (15–17). We also addressed computationally whether the introduced changes would have major impacts over RNA secondary structure using the mfold web server (22) and found that the predicted secondary structure for Stop and SuperStop did not considerably change (data not shown).

Using these viral stocks, we compared replication kinetics and RNA synthesis in standard BHK-21 (hamster) and C6/36 (Aedes albopictus) cell lines at a multiplicity of infection (MOI) of 0.1 PFU per cell. During the first viral replication cycle (up to 8 h postinfection [hpi]), production of infectious virus was very close for variants compared to that of the wild type. In subsequent cycles, Stop and SuperStop produced less infectious virus than the wild type (Fig. 1D and G). This phenotype was not related to defects in RNA synthesis since genome copy numbers were similar among viruses (Fig. 1E and H). We hypothesize that after the initial replication cycle, the introduction of stop codons or other mutations in the RNA of the Stop and SuperStop viruses reduced the amount of infectious virus produced. This is supported by the lower specific infectivity observed for Stop and SuperStop at 48 hpi than for the wild type (Fig. 1F and I).

**FIG 2:**
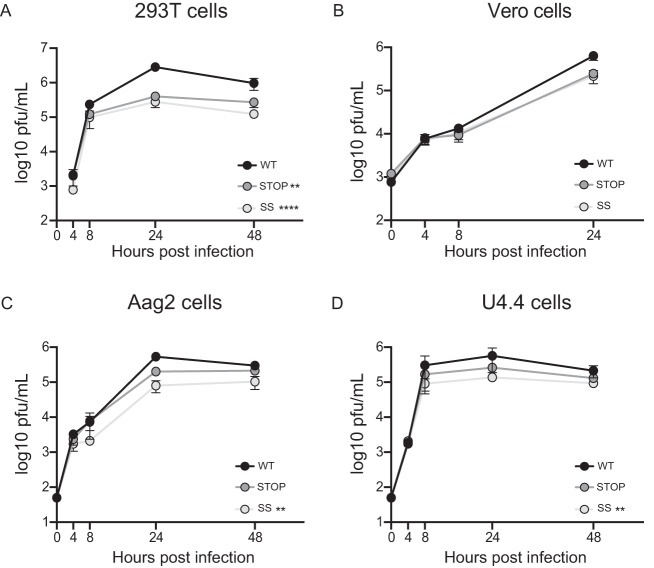
Replication kinetics in mammalian and insect cell lines. Replication kinetics of WT, Stop, and SS viruses at an MOI of 0.1 PFU/cell in 293T (A) and Vero (B) mammalian cell lines and in Aag2 (C) and U4.4 (D) insect cells. Mean virus titers and standard errors of the means are shown (*n* = 3). **, *P* < 0.01; ****, *P* < 0.0001 (by 2-way analysis of variance with Tukey’s multiple-comparison test). For all cases, no statistical significance is indicated if *P* is >0.05.

To expand these observations, we determined virus titers in other cell lines relevant to CHIKV infection. Given that the virus alternates between hosts, we selected 293T (human) and Vero (monkey) cells to evaluate replication in the mammalian host and in Aag2 (Aedes aegypti) and U4.4 (Aedes albopictus) cells for the insect counterpart. Multistep growth kinetics were carried out at an MOI of 0.1 PFU/cell. Again, while kinetics were similar during the first replication cycle (first 8 h), in all cases the Stop and SuperStop viruses replicated with lower titers than the wild type during subsequent replication cycles (Fig. 2A to D).

### CHIKV variants are more susceptible to antiviral treatment and present smaller plaque sizes.

**FIG 3:**
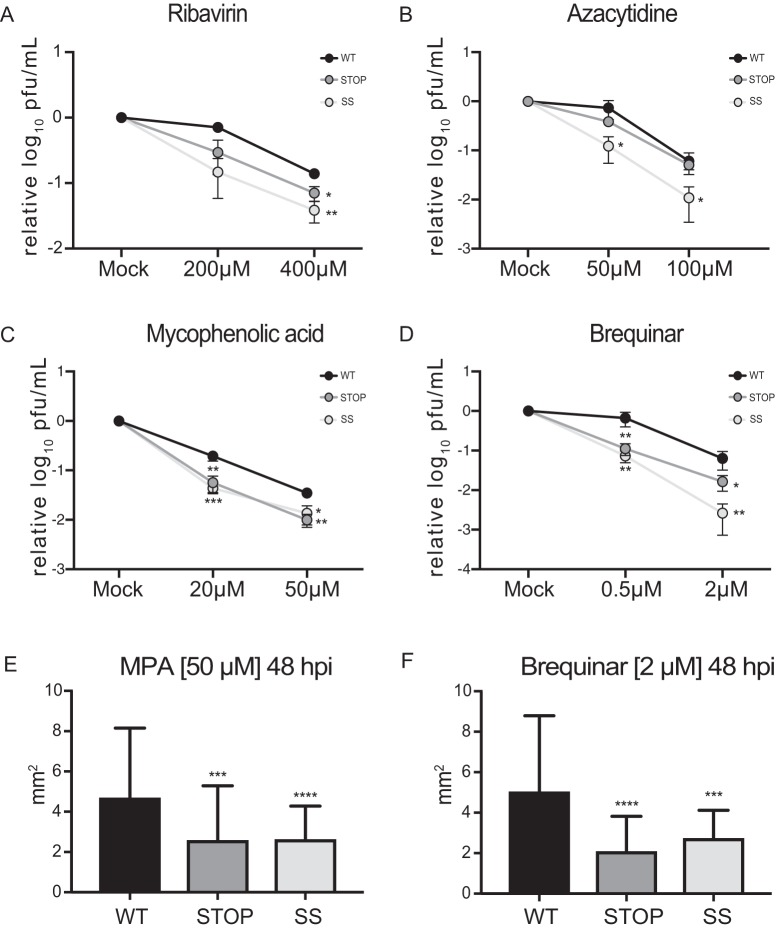
Stop and SuperStop viruses are more sensitive to antiviral treatment and have decreased fitness. (A to D) Cells infected at an MOI of 0.1 PFU/cell with WT, Stop, and SS viruses were treated with increased concentrations of ribavirin, azacytidine, mycophenolic acid (MPA), or brequinar, as indicated. Mean virus titers and standard errors of the means are shown (*n* = 3). Significance is indicated as follows: *, *P* < 0.0332; **, *P*< 0.0021; ***, *P* < 0.0002 (by an unpaired two-tailed *t* test). (E and F) Plaque size was determined for viral supernatants treated for 48 h with a high dose of MPA or brequinar. Mean plaque size and standard errors of the mean are shown (*n* > 34). Significance is indicated as follows: ***, *P* = 0.0008; ****, *P* < 0.0001 (MPA); ***, *P* = 0.0003; ****, *P* < 0.0001 (brequinar) (by one-way analysis of variance with Tukey’s multiple-comparison test). Unless noted otherwise, differences were not statistically significant (*P* > 0.1234).

Next, we determined the sensitivity to different drug treatments and the viral fitness of Stop and SuperStop viruses versus that of the wild type. We chose two base analogs, ribavirin and azacytidine (AZC), that induce an overall mutagenic effect (23, 24). We subjected viral populations to a mock infection (MOI of 0.1 PFU/cell) or to increasing doses of each drug, and 48 h after infection supernatants were collected. Viral titers were determined and expressed relative to those of mock conditions. We found that the less robust virus, SuperStop, is more sensitive to treatment than the Stop virus, which is more sensitive than the wild type (Fig. 3A and B). Next, we tested the viruses against antiviral drugs that are known to inhibit RNA virus replication: brequinar and mycophenolic acid (MPA). They block *de novo* pyrimidine biosynthesis, depleting the intracellular pyrimidine pool (25), and also induce the innate immune response (26, 27). For all cases, Stop and SuperStop viruses were significantly more susceptible to antiviral treatment in a dose-dependent manner (Fig. 3C and D). We also determined plaque size as a proxy of viral fitness. Supernatants, collected at 48 h postinfection under high-dose treatment, were used for a plaque phenotype assay. Again, Stop and SuperStop viruses exhibited significantly smaller plaque sizes under all conditions, indicating that these viral populations had lower fitness than the wild type (Fig. 3E and F).

### CHIKV Stop and SuperStop viruses are attenuated *in vivo*.

**FIG 4:**
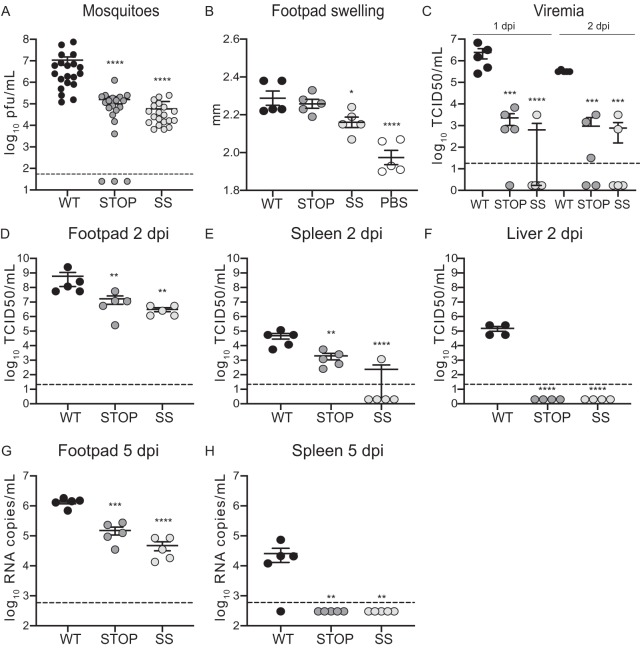
Stop and SuperStop viruses are attenuated *in vivo*. Aedes albopictus mosquitoes and wild-type C57BL/6 mice were infected with WT, Stop, and SS viruses. Mean values and standard errors of the means are shown. Horizontal dashed lines indicate the limit of detection of the assay. (A) Viral titers in mosquitoes at 10 days postinfection (*n* = 20). ****, *P* < 0.0001 (by a Mann-Whitney two-tailed unpaired *t* test). (B) Left footpad swelling at 2 days postinfection (*n* = 5). *, *P* = 0.0327; ****, *P* < 0.0001 (by one-way analysis of variance with Tukey’s multiple-comparison test). (C) Viremia at days 1 and 2 postinfection (*n* = 5). ***, *P* = 0.0003; ****, *P* < 0.0001 (1 dpi); Stop ***, *P* = 0.0011; SS ***, *P* = 0.0004 (2 dpi) (by one-way analysis of variance with Tukey’s multiple-comparison test). (D to F) Viral titers in footpad, spleen, and liver at 2 days postinfection. Titers were determined after an amplification cycle in C6/36 cells (*n* = 5). Stop **, *P* = 0.0084; SS **, *P* = 0.0015 (panel D); **, *P* = 0.0084; ****, *P* < 0.0001 (panel E); ****, *P* < 0.0001 (F) (by one-way analysis of variance with Tukey’s multiple-comparison test). (G and H) Viral RNA copy numbers in footpad and spleen at 5 days postinfection (*n* = 5). **, *P* = 0.0015; ***, *P* = 0.0006; ****, *P* < 0.0001 (by one-way analysis of variance with Tukey’s multiple-comparison test). For all cases, no statistical significance is indicated if *P* is >0.05.

To evaluate the effects of codon replacement in CHIKV in *in vivo* models and to determine whether viruses were attenuated, we started by infecting Aedes albopictus mosquitoes. A blood meal containing 10^6^ PFU of wild-type virus or variants was given to 20 mosquitoes, and viral titers were determined in full bodies at 10 days postinfection (dpi) (Fig. 4A). Mosquitoes infected with CHIKV variants contained significantly fewer infectious viral particles, indicating attenuation for both Stop and SuperStop viruses.

We next evaluated infection in the mammalian model. We infected C57BL/6 mice with 10^4^ PFU of wild-type virus or variants in the left footpad (*n* = 5 per group). Blood was collected at 1 and 2 days after infection, while footpad swelling was measured at 2 days, after which target organs were harvested. We observed significantly decreased footpad swelling following infection with the SuperStop virus compared to that with wild-type virus infection, indicating a milder inflammatory state (Fig. 4B). Viremia on both days was significantly lower with the variants than with the wild type. In fact, some mice in both Stop and SuperStop groups did not present any detectable viremia (Fig. 4C). In addition, we observed 2-log- and 3-log-lower virus titers for Stop and SuperStop, respectively, at the site of injection, indicating decreased replicative capacities for these viruses already at the primary site of replication (Fig. 4D). This strong attenuation was also observed in the spleen, where in the case of SuperStop virus, no viable viral particles were recovered for 4 out of 5 mice (Fig. 4E). Moreover, we did not recover any virus in the liver of mice infected with Stop and SuperStop, while considerable replication for wild-type virus was observed (Fig. 4F). The data shown in Fig. 4D to F corresponding to titers in footpads, spleen, and liver were determined after an amplification cycle in C6/36 cells, as described previously (28). To exclude the possibility that the observed differences were due to delayed replication kinetics in C6/36 cells (Fig. 1G), the amplification step was also performed in BHK-21 cells (where replication kinetics are not statistically different) (Fig. 1D), and the same results were obtained (data not shown). Taken together, these results point to significant attenuation for Stop and SuperStop viruses and reduced capacity to disseminate in adult wild-type-infected mice, particularly for SuperStop virus. In order to determine whether the observed effect was due to an intrinsic defect of the virus in crossing anatomical barriers and colonizing organs or simply due to slower replication kinetics, we extended the experiment to 5 days after infection, allowing the virus more time to replicate and disseminate. No infectious virus was recovered for any virus in any organ (data not shown). Regardless, we were able to measure viral RNA through reverse transcription-quantitative PCR (RT-qPCR). We found that viral RNA was completely cleared from the liver for all three groups (data not shown). In footpad, we observed significantly lower levels of viral RNA in Stop- and SuperStop-infected mice than in wild-type-infected mice (Fig. 4G). In the case of spleen, viral RNA was detected only in wild-type-infected mice but not in mice infected with other viruses (Fig. 4H).

**FIG 5:**
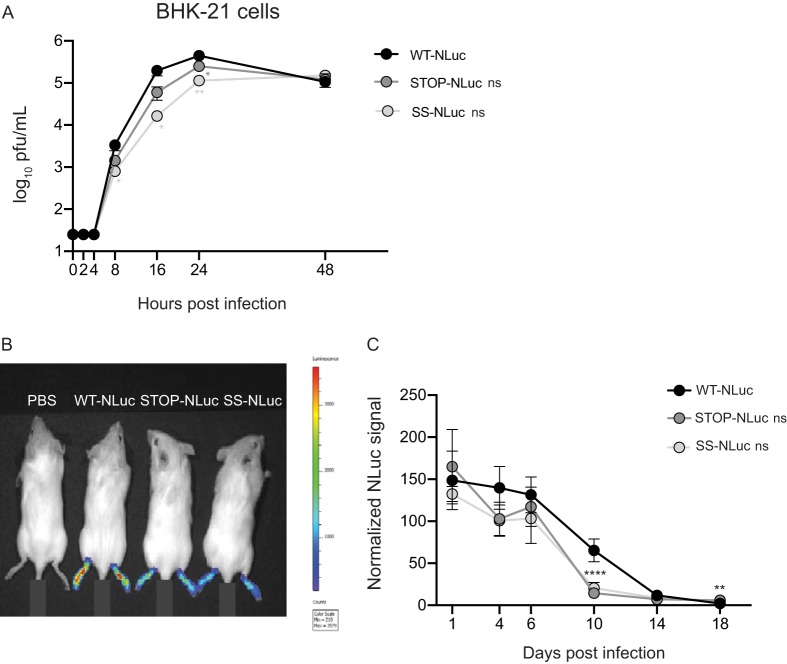
Stop and SuperStop virus *in vivo* kinetics followed by live imaging. Wild-type albino C57BL/6 mice were infected in the left footpad with WT, Stop, and SS viruses expressing nano-luciferase (WT-NLuc, Stop-NLuc, and SS-NLuc, respectively), and replication kinetics were followed over time by *in vivo* imaging. (A) Replication kinetics of WT-NLuc, Stop-NLuc, and SS-NLuc in BHK-21 cells. Mean relative virus titers with standard errors of the means are shown (*n* = 3). Significance is indicated for individual time points as follows: 8 hpi SS-NLuc *, *P* = 0.0345; 16 hpi SS-NLuc *, *P* = 0.0192; 24 hpi Stop-NLuc *, *P* = 0.0170; 24 hpi SS-NLuc **, *P* = 0.0083; 48 hpi, no significance (ns), *P* > 0.05 (by two-way analysis of variance with Tukey’s multiple-comparison test). (B) Representative image of the experiment (pilot study) including mice infected with a PBS control, WT-NLuc, Stop-NLuc, and SS-NLuc in both footpads and imaged at 2 dpi. (C) NLuc values normalized by the value of the negative control measured on the left footpads of mice infected with WT-NLuc, Stop-NLuc, and SS-NLuc at the indicated time points Mean values with standard errors of the means are shown (*n* = 5). ****, *P* < 0.0001 (Stop and SS); **, *P* = 0.0018 (Stop). To improve homoscedasticity of the data, statistics were performed using log-transformed data (two-way analysis of variance with Tukey’s multiple-comparison test).

Finally, we generated viruses expressing nano-luciferase (NLuc) to evaluate the kinetics of infection through live imaging over time. Initially, we determined replication kinetics of wild-type, Stop, and SuperStop viruses expressing NLuc (WT-NLuc, Stop-NLuc and SuperStop-NLuc, respectively) in BHK-21 cells (Fig. 5A). We found that while there is no statistical significance for overall replication kinetics in NLuc-expressing viruses, we observed a defect in replication at early time points for Stop-NLuc and SuperStop-NLuc (8 hpi, 16 hpi, and 24 hpi), which was no longer observed at 48 hpi.

Next, we infected C57BL/6 albino mice with 5 × 10^4^ PFU of each NLuc-expressing virus in the left footpad (*n* = 5 per group), and at days 1, 4, 6, 10, 14, and 18 postinfection, mice were anesthetized and administered the NLuc substrate (furimazine) via intraperitoneal injection. After 10 min, groups of mice were imaged using a Tecan IVIS Spectrum imaging device. NLuc was quantified on each left footpad and normalized to the level of the negative control for each group and time point. For comparative reasons, in Fig. 5B an image of our pilot study is shown, where one mouse per group was imaged at the same time at 2 days postinfection. While the signal for the negative-control group (inoculated with phosphate-buffered saline [PBS]) was undetectable, the intensity of signal was highest for the wild-type CHIKV-NLuc-injected mouse and decreased accordingly for the Stop-NLuc and SuperStop-NLuc viruses. Of note, in this pilot study we injected both footpads, while in further experiments we injected only the left one. In Fig. 5C the results for replicating NLuc-expressing viruses over time are shown, where NLuc signal was normalized and plotted for all three groups at each time point. The signals for Stop-NLuc and SuperStop-NLuc were lower than those of the wild-type CHIKV-NLuc at 4, 6, and 10 days after infection, indicating reduced replication over time. Notably, the normalized signal at 10 days postinfection was close to 0 for Stop-NLuc and SuperStop-NLuc and significantly lower than that of the wild type, indicating faster clearance of the virus.

### Stop and SuperStop viruses can induce neutralizing antibodies that protect against challenge infection.

**FIG 6:**
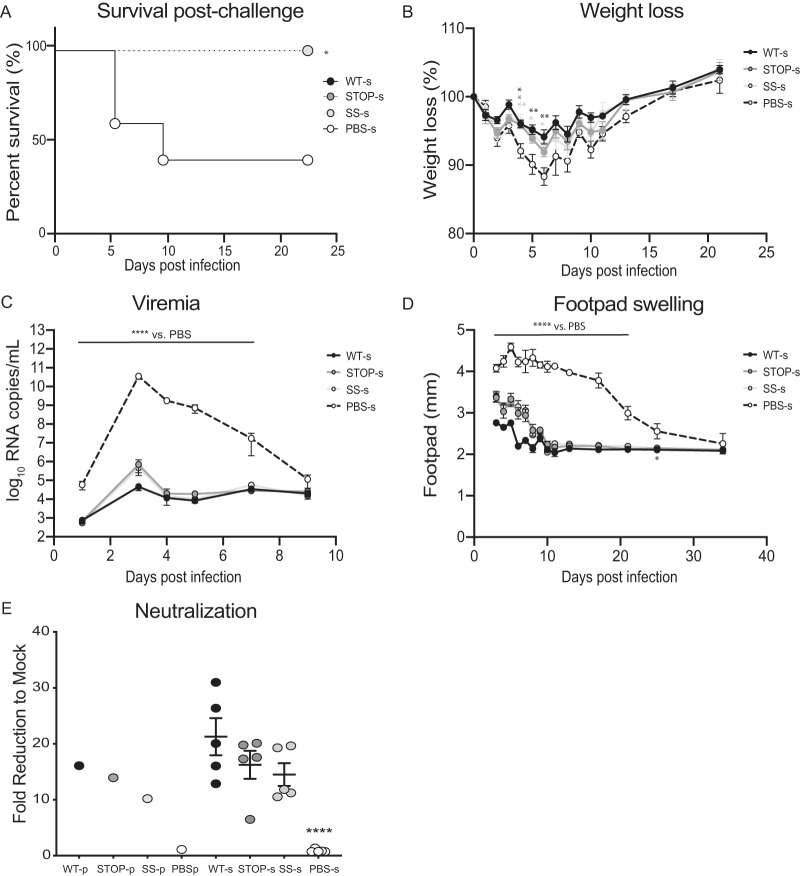
Stop and SuperStop protect against challenge infection. Sera from mice infected with WT, Stop, or SS virus or mock infected with PBS (WT-s, Stop-s, SS-s, or PBS-s) were transferred to C57BL/6 mice that had previously received type I IFN-α/β receptor antibody and were challenged with WT CHIKV. (A) Survival after challenge of mice (*n* = 5). *, *P* = 0.0116 (Kaplan-Meyer with Mantel-Cox test). (B) Weight change after challenge. Values are means with standard errors of the means (*n* = 5). Day 4 WT-s *, *P* = 0.0174; day 4 Stop-s *, *P* = 0.0218; day 4 SS-s **, *P* = 0.098; day 5 WT-s **, *P* = 0.0068; day 5 SS-s *, *P* = 0.0198; day 6, WT-s **, *P* = 0.0016; day 6 SS-s *, *P* = 0.0187 (two-way analysis of variance with Tukey’s multiple-comparison test). (C) Viremia was measured at days 1, 3, 4, 5, 7, and 9 postchallenge. Values are means with standard errors of the means (*n* = 5). For days 1, 3, 4, 5 and 7, values for WT-s, Stop-s, and SS-s were significantly different from the value for PBS-s (****, *P* < 0.0001) (two-way ANOVA with Tukey’s multiple-comparison test). (D) Footpad swelling at days 3, 4, 5, 6, 7, 8, 9, 10, 11, 13, 17, 21, 25, and 34 postchallenge. Values are means with standard errors of the means (*n* = 5). Values for WT-s, Stop-s, and SS-s were significantly different from the value for PBS-s at days 3, 4, 5, 6, 7, 8, 9, 10, 11, 13, 17, and 21 (****, *P* < 0.0001) and at day 25 (*, *P* < 0.05) (two-way analysis of variance with Tukey’s multiple-comparison test). (E) Neutralizing antibody titers for individual serum samples and pooled (p) sera (used in passive transfer) from mice infected with WT (WT-p), Stop (Stop-p), and SS (SS-p) virus and mock infected with PBS (PBS-p). Values are means with standard errors of the means (*n* = 5). Differences in the values for Stop-s (*P* = 0.3210) and SS-s (*P* = 0.1335) were nonsignificant (*P* > 0.05) relative to values for WT-s. ****, *P* < 0.0001 (one-way analysis of variance with Tukey’s multiple-comparison test). For pooled sera (*n* = 1), no statistical tests were performed.

Given the attenuation observed in both hosts, our next goal was to determine the immunogenic potential of these viruses and evaluate their protective capacity against challenge infection. We immunized 5-week-old C57BL/6 mice with 10^4^ PFU of wild-type, Stop, or SuperStop virus via footpad injection. After 4 weeks, sera containing neutralizing antibodies were collected, heat inactivated, and passively transferred to naive mice, which had previously received a dose of type I interferon (IFN) receptor antibody. Naïve mice receiving this antibody are more susceptible to infection and develop severe disease or succumb to infection when challenged with CHIKV. The passive transfer of sera from wild-type (WT-s), Stop (Stop-s), and SuperStop (SS-s) groups protected all mice from lethality. In the negative-control group where mice received only the type I IFN receptor antibody (no virus challenge), we also observed 100% survival (data not shown). On the other hand, in the group that received sera generated from a PBS mock immunization (PBS-s), all mice showed signs of disease, and 3 out of 5 succumbed to CHIKV infection (Fig. 6A). Morbidity was also monitored by weight change. Mice in the WT-s and SS-s groups lost significantly less weight than the PBS-s group at days 4, 5, and 6, indicating protection against infection. Mice in the Stop-s group lost significantly less weight than those in the PBS-s group at day 4 postchallenge (Fig. 6B). Viremia was measured at 1, 3, 4, 5, 7, and 9 days postchallenge and showed significant differences for all groups versus the PBS-s-immunized control group (Fig. 6C). This indicates that virus production and dissemination are being controlled by the presence of neutralizing antibodies presumably transferred from sera from wild-type-, Stop- and SuperStop-immunized mice. Footpad swelling, an indicator of inflammation and proxy of local viral replication, showed sustained and significantly higher values for the PBS-s group than for the WT-s, Stop-s, and SS-s groups up to 21 days after challenge (for the PBS-s mice that survived). At early time points Stop-s and SS-s groups exhibited increased footpad swelling compared to that of the WT-s group, indicating that the afforded protection is not as strong as that with a wild-type infection (Fig. 6D). Finally, we measured the neutralizing antibody titers present in each individual serum sample and in the pooled sera we passively transferred. Neutralizing antibody levels, expressed as fold reduction relative to the level of mock treatment, were significantly higher than those in PBS sera, and no significant differences between the levels of the wild-type, Stop, and SuperStop groups were detected (Fig. 6E).

### Stop and SuperStop viruses can be transmitted from Aedes aegypti mosquitoes to neonatal mice, but their replication is deficient in the mammalian host.

**FIG 7:**
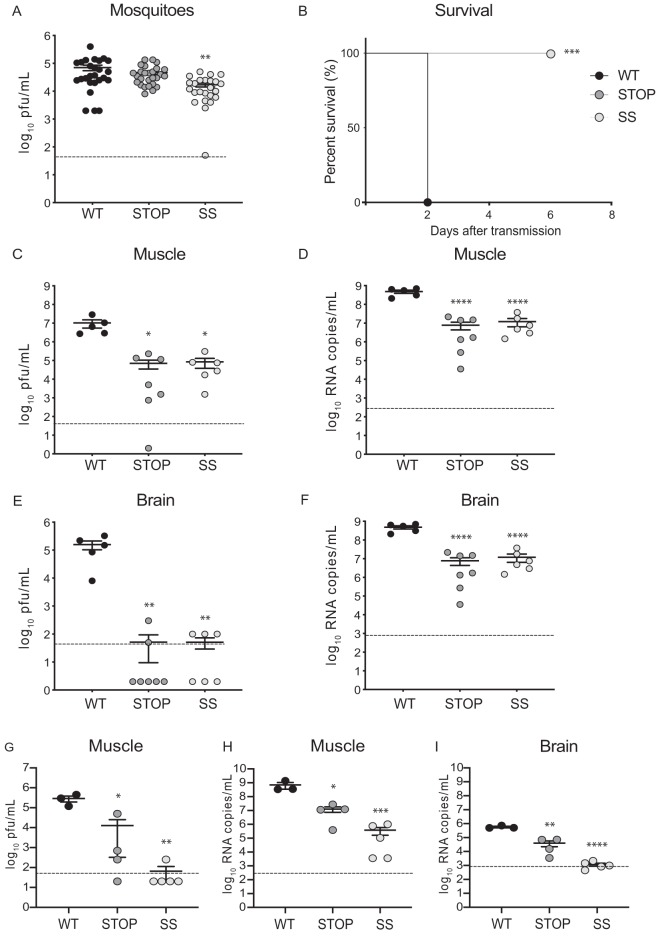
Stop and SuperStop viruses are transmitted from mosquitoes to mice, resulting in very mild infection. Aedes aegypti mosquitoes were infected with WT, Stop, or SS virus and after 14 days put in contact with neonatal mice to allow feeding and transmission of virus. Two days (WT) or 6 days (Stop and SS virus) after transmission, target tissues were harvested for virus quantification by PFU counts and genome copy numbers. Values are means with standard errors of the means. Horizontal dashed lines indicate the limit of detection. (A) Viral titers in mosquitoes at 14 days postinfection (WT, *n* = 26; Stop, *n* = 27; SS, *n* = 24). **, *P* = 0.0018 (one-way analysis of variance with Tukey’s multiple-comparison test). (B) Percent survival of mice after transmission (*n* = 5 for WT, *n* = 7 for Stop, and *n* = 6 for SS). ***, *P* = 0.0002 (Kaplan-Meyer with Mantel-Cox test). (C and D) Viral titers and RNA genome copy numbers in leg muscle (*n* = 5 for WT, *n* = 7 for Stop, and *n* = 6 for SS). Stop *, *P* = 0.0127; SS *, *P* = 0.0157; ****, *P* < 0.0001 (one-way analysis of variance with Tukey’s multiple-comparison test). (E and F) Viral titers and RNA genome copy numbers in brains of mice (*n* = 5 for WT, *n* = 7 for Stop, and *n* = 6 for SS). Stop **, *P* = 0.0018; SS **, *P* = 0.0024; ****, *P* < 0.0001 (one-way analysis of variance with Tukey’s multiple-comparison test). (G to I) Neonatal mice were infected subcutaneously; at 6 days postinfection mice were euthanized, and target tissues were collected (*n* = 3 for WT, *n* = 4 for Stop, and *n* = 5 for SS). Significance was determined by one-way analysis of variance with Tukey’s multiple-comparison test. Viral titers (G) and genome copy numbers (H) were determined in leg muscle of mice. *, *P* = 0.0105; ***, *P* = 0.0006 (panel G); *, *P* = 0.0351; ***, *P* = 0.0009 (panel H). Virus genome copy numbers were determined in brains of mice (I). **, *P* = 0.0052; ****, *P* < 0.0001. Unless otherwise stated, differences were not statistically significant (*P* < 0.05).

Next, we evaluated the transmissibility of these viruses in an experimental setting that more closely resembles what occurs in the wild using a transmission model (29). To do this, we used Aedes aegypti mosquitoes, the other relevant mosquito species for CHIKV transmission, and neonatal C57BL/6 mice that are more susceptible than adult mice to infection, given that their immune systems have not fully developed. We infected mosquitoes with viruses using an artificial blood meal containing 10^6^ PFU/ml. Transmission was performed at day 14 by allowing each group of infected mosquitoes to feed on neonatal mice (*n* = 5 for the wild-type group, *n* = 7 for the Stop group, and *n* = 6 for the SuperStop group), after which full mosquitoes were homogenized, and titers were determined. We found significantly lower titers in SuperStop-infected mosquitoes than in wild-type-infected insects (Fig. 7A). Following transmission, neonatal mice were monitored for survival and limb paralysis and euthanized at 6 days after transmission; target tissues were collected, and viral load (infectious titers and genomic RNA levels) was determined. In terms of survival, 4 out of 5 mice in the wild-type-infected group succumbed to infection by day 2 after transmission, and the remaining one had to be euthanized due to limb paralysis on the same day, while all mice in the Stop and SuperStop infection groups survived until the end of the experiment (Fig. 7B). Mortality was statistically significant for Stop- and SuperStop-infected mice compared to that of wild-type-infected mice.

The results for brains and leg muscles of mice infected by transmission of virus are plotted in Fig. 7C to F. It is worth noting that, in these panels, we are comparing the results obtained in mice at day 2 for wild-type transmitted virus and at day 6 for Stop and SuperStop transmitted viruses. When performing the experiment, we favored survival measurements and therefore kept Stop- and SuperStop-infected mice alive until the end of the experiment (6 dpi), even when wild-type-infected mice had died (at 2 dpi). For this reason, comparison of viral particles and genome copy numbers for brains and leg muscles are made at different time points for different groups. Our findings show that after transmission through mosquito bite, Stop and Superstop are significantly attenuated both in terms of viral particles and genome copy numbers compared to levels in in brain and leg muscle of mice infected by transmission of wild-type CHIKV. Interestingly, we recovered very few viral particles in the brains of some Stop- and SuperStop-infected mice, pointing either to a defect in their dissemination capacity or to less infectious virus being produced, given that viral RNA may have already incorporated a significant number of stop codons. These results are similar to those observed in a previous transmission cycle we performed, where no viral particles were recovered in the brains of SuperStop-infected mice (data not shown).

More striking results were obtained when wild-type, Stop, and SuperStop viruses were directly inoculated subcutaneously in neonatal mice (Fig. 7G to I). After 6 days postinfection, in leg muscles we observed significantly decreased viral titers for Stop and SuperStop viruses (PFU recovered in only 1 mouse for SuperStop) (Fig. 7G). RNA levels for Stop- and SuperStop-infected mice were significantly lower than those for wild-type infection (Fig. 7H). No infectious particles were detected in the brain of any mouse (data not shown), whereas RNA genomes were detected for wild-type-infected and some Stop-infected mice but were either negative or very close to the lowest limit of detection for SuperStop-infected mice (Fig. 7I).

### Stop and SuperStop viruses introduce more stop codons in the insect and mammalian host.

**FIG 8:**
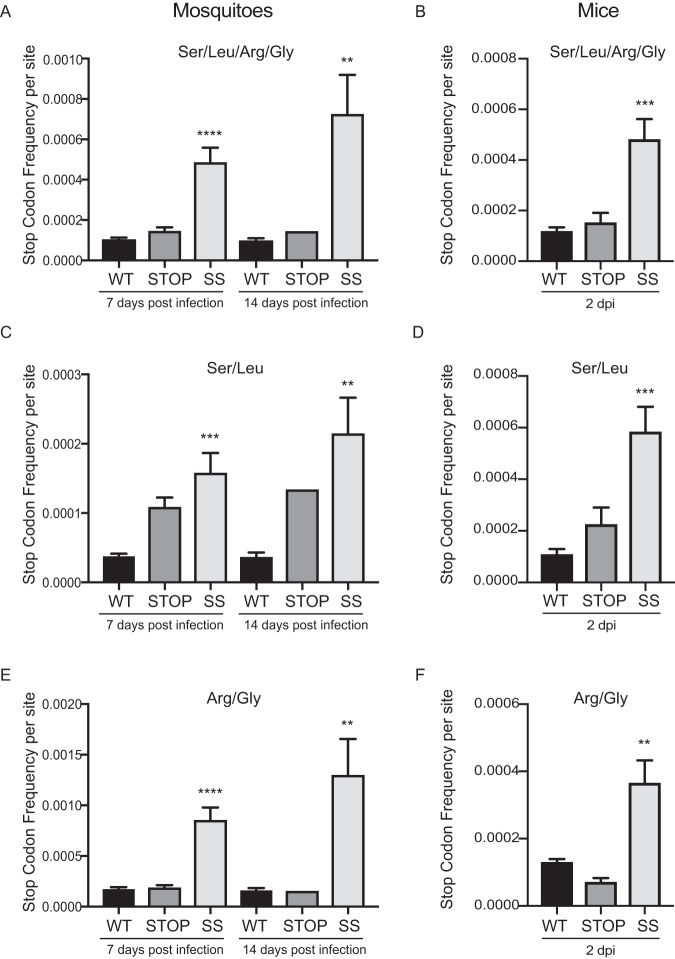
Stop and SuperStop viruses introduce more stop codons in the insect and mammalian hosts. From Illumina sequencing reads, we determined the stop codon frequency per site for positions where synonymous codons were replaced in WT, Stop, and SS viruses in whole mosquitoes infected at 7 and 14 days postinfection and from footpads of infected mice at 2 days postinfection. Values are means and standard errors of the means. Significance was determined by one-way analysis of variance with Tukey’s multiple-comparison test. (A) Stop codon frequency per site at all positions where synonymous codons were replaced, as determined in infected mosquitoes at days 7 and 14 postinfection (*n* = 8 for WT and SS and *n* = 5 for Stop at 7 dpi; *n* = 6 for WT, *n* = 1 for Stop, and *n* = 3 for SS at 14 dpi). ****, *P* < 0.0001; **, *P* = 0.0033. (B) Stop codon frequency per site where all synonymous codons were replaced, as determined in infected footpads of mice at day 2 days postinfection (*n* = 5). ***, *P* = 0.0009. (C) Stop codon frequency where synonymous codons for serine and leucine were replaced, as determined in infected mosquitoes at days 7 and 14 postinfection (*n* = 8 for WT and SS and *n* = 5 for Stop at 7 dpi; *n* = 6 for WT, *n* = 1 for Stop, and *n* = 3 for SS at 14 dpi). ***, *P* = 0.0007; **, *P* = 0.0016. (D) Stop codon frequency in positions where synonymous codons for serine and leucine were replaced, as determined in infected footpads at 2 days postinfection (*n* = 5). ***, *P* = 0.0009. (E) Stop codon frequency in positions where synonymous codons for arginine and glycine were replaced, as determined in infected mosquitoes at days 7 and 14 postinfection (*n* = 8 for WT and SS and *n* = 5 for Stop at 7 dpi; *n* = 6 for WT, *n* = 1 for Stop, and *n* = 3 for SS at 14 dpi). ****, *P* < 0.0005; **, *P* = 0.0022. (F) Stop codon frequency in positions where synonymous codons for arginine and glycine were replaced, as determined in infected footpads at 2 days postinfection (*n* = 5). **, *P* = 0.0031. Unless otherwise stated, differences were not statistically significant (*P* < 0.05), with the exception of Stop virus infection of mosquitoes at 14 dpi (*n* = 1, due to death or noninfection of mosquitoes; no statistical tests were performed).

Given that this genomic design relies on viruses having a higher chance of incorporating stop codons, we quantified the number of stop codons present in infected mosquitoes and footpads from infected mice. We generated amplicon-based libraries covering the structural region of the genome of all samples and sequenced them using Illumina technology. From the reads obtained, we interrogated the positions we modified in our genomic design (codons for Leu, Ser, Arg, and Gly), and we determined the stop codon frequency per site (SCF/site) for each sample. Our analysis confirmed that the Stop and SuperStop viruses incorporated more stop codons in their progeny genomes than the wild type both in mosquitoes (Fig. 8A) and in mouse footpads (Fig. 8B). Interestingly, when analyzing the SCF/site considering only Leu/Ser positions (replaced in Stop and SuperStop viruses) (Fig. 8C and D) or Arg/Gly positions (replaced only in the SuperStop virus) (Fig. 8E and F), we observed inverse patterns. In mosquito samples the majority of stop codons were introduced at the Arg/Gly-substituted positions (Fig. 8E), with minimal contribution from Ser/Leu positions (Fig. 8C). On the other hand, in the mammalian samples the majority of stop codons were introduced at the Ser/Leu-substituted positions (Fig. 8D), with a minimal contribution from Arg/Gly-replaced positions (Fig. 8F).

## DISCUSSION

In the recent years CHIKV has turned into a major health problem worldwide, given its rapid expansion (30), the number of people at risk given the geographical distribution of the mosquito vectors Aedes aegypti and Aedes albopictus (31–33), and its potentially increased virulence in humans (34). Accordingly, CHIKV might be mimicking what has been observed during dengue virus evolution by perpetuating an enzootic-independent cycle with high incidence in the human population (11, 34, 35). Therefore, it has become imperative to find efficient vaccines and treatments. Indeed, some CHIKV vaccine candidates have been proposed, and some have reached clinical trials (reviewed in reference 8). These include inactivated vaccines, which despite being safe, present challenges in efficiency, production, and costs that may make them inaccessible, especially for the low-resource countries where CHIKV is endemic. In contrast, LAVs are lower cost, relatively easy to produce, and generally confer long-lasting immunity but have as a disadvantage the risk of reversion to pathogenic phenotypes (36). In this study, we followed a strategy previously described by our lab (18) to develop CHIKV LAV candidates. This rational approach involves reducing the mutational robustness of CHIKV by introducing hundreds of synonymous codon replacements that render the viruses more intolerant to mutations.

We generated two LAV candidates, named the Stop (replacements for Ser and Leu) and the SuperStop (replacements for Ser, Leu, Arg, and Gly) viruses. When the viruses were evaluated in both the insect and the mammalian host, we found that they were strongly attenuated. The attenuated phenotype was stronger for the SuperStop virus, which carries more codon substitutions. A clear example of attenuation for our candidate LAVs is the reduced or absent viremia in Stop- and SuperStop-infected mice and the decreased dissemination to the liver and most spleens of SuperStop-infected mice, which may be a consequence of the absent viremia (Fig. 4C, E, and F). When following infection over time through *in vivo* imaging, we noticed faster clearance from the mammalian host (Fig. 5C). We speculate that our vaccine candidates have a defect in crossing anatomical barriers and colonizing organs. This defect may be due to their decreased mutational robustness, resulting in lower viral progeny and smaller population size; or, possibly, the codon changes no longer permit adaptive mutations required to overcome host bottlenecks, a defect in evolvability (18, 19).

We also examined *in vivo* the immunogenicity and protective efficacy of the mutant viruses in order to determine whether these could be effective LAV candidates that could protect the host from CHIKV exposure. This was done by passive transfer of sera containing neutralizing antibodies to mice that had received the type I IFN-α/β receptor antibody, which renders mice more susceptible to infection. We found that mice that received Stop and SuperStop transferred sera survived challenge, had decreased or absent viremia, and developed greatly diminished disease symptoms characterized only by discrete footpad swelling and mild weight loss (Fig. 6A to D). Yet in some experiments small differences were observed compared to symptoms in wild-type-vaccinated mice, possibly related to the slightly lower titers in neutralizing antibodies (nonsignificant) (Fig. 6E). It is possible that, given that the codon changes are introduced in the structural open reading frame of the CHIKV genome where most important epitopes are present, the new evolutionary trajectories of the replaced codons may have disrupted some of them (particularly envelope proteins) and, therefore, yielded different or new targets for neutralizing antibodies (37–39).

In order to evaluate potential drawbacks related to the amplification and transmission in the two hosts, we assessed the LAV candidates in their natural transmission cycle. We found that Stop and SuperStop can be transmitted through mosquito bite, with development of only an asymptomatic or transient infection. Mice bitten by wild-type-infected mosquitoes succumbed to infection by day 2 after transmission, while mice bitten by either Stop- or SuperStop-infected mosquitoes survived until the end of the experiment with no apparent signs of disease (Fig. 7B). Remarkably, we recovered viral particles in the brains of only a few infected mice and at very low titers (Fig. 7E and F), pointing either to a defect in dissemination or to decreased production of infectious viral particles, given that viral RNA may have incorporated too many detrimental stop codons. Indeed, given that the stop codon count is significantly higher for SuperStop virus in mosquitoes and mice (Fig. 8A and B), we believe that a considerable proportion of viral RNA may be (almost) noninfectious, accounting for the attenuated phenotype and compromised transmissibility and dissemination.

One interesting point raised from the stop codon frequency analyses is the different profiles of stop codon incorporation in the two hosts. While in mosquitoes arginine and glycine 1-to-stop codons seem to contribute the most to the overall stop codon count, in mice, we observed the opposite, with serine and leucine 1-to-stop contributing the most. We find this mutational bias to be very interesting, but it remains to be explored in future research.

All in all, we propose that even though both viruses may have potential as novel vaccine candidates against CHIKV, the Superstop virus is the better suited one. It presents very strong attenuation levels in both hosts, rapid clearance, very low to undetectable viremia that prevents mosquitoes from acquiring the virus if they bite a vaccinated individual, and reduced inflammation at the site of injection. It confers protection against challenge infection after a single dose, has decreased transmissibility, as observed during a transmission cycle from mosquitoes to mice, and, importantly, has a significantly higher stop codon count in both hosts, which confirms the rational genomic design (synonymous codon replacement of serine, leucine, arginine, and glycine). Additionally, this type of LAV overcomes one of the main concerns related to LAVs, which is the reversion to pathogenic phenotypes. Since the attenuation results from so many changes in synonymous codons contributing equally, the risk of reversion to a pathogenic phenotype, even after several rounds of replication, is unlikely. No single codon change is dominant; rather, each of the over 200 changes contributes just a little to the overall attenuation (similar to the approach “death by a thousand cuts” as proposed by the Mueller lab [13]). Indeed, even after 14 days of infection in Aedes albopictus and Aedes aegypti mosquitoes, reversion was not observed.

An important consideration requiring considerable future effort would be determining the effects of the swapping codons on volatility (the probability of introducing a nonsynonymous change after point mutation [40]) and evolvability (the ability to acquire adaptive mutations). It is possible that in addition to stop mutations, the altered codons could result in new amino acids being introduced that have the unwanted effect of improving virus fitness.

In applying our genomic design to yet another RNA virus family (18), we confirmed that decreasing mutational robustness could be employed as a universal tool for attenuating RNA viruses. Yet the stronger phenotype obtained in alphaviruses when the same genomic design as used in picornaviruses and orthomyxoviruses is applied indicates that altering mutational robustness has different impacts, possibly related to the biology of each viral family. Because they alternate between two hosts, alphaviruses are subjected to stronger purifying selection (41–44), given that any mutation that confers a fitness advantage in one host could be detrimental in the other host (45–47). We think that CHIKV and possibly most arboviruses have evolved strong mutational robustness compared to that of other RNA viruses in order to cope with the cycling lifestyle. In being mutationally robust (buffering the effects of mutations), CHIKV is able to reach a balance between the mutation load, selection, and different replication environments. We believe that in this study, by decreasing mutational robustness, we significantly affected the evolutionary strategies developed by CHIKV for dual-host replication, resulting in a significantly stronger phenotype and attenuation than with previous results in other RNA viruses (18), rendering these virus strains better vaccine candidates.

## MATERIALS AND METHODS

### Cells and viruses.

Mammalian Vero, 293T, and BHK-21 cell lines were maintained in Dulbecco’s modified Eagle’s medium (DMEM; Thermo Fisher) supplemented with 10% fetal bovine serum (FBS; Thermo Fisher) and 1% penicillin-streptomycin (P-S; Thermo Fisher). Cells were maintained at 37°C in 5% CO_2_. Mosquito cell lines C6/36, U4.4, and Aag2 were grown in Leibovitz medium (L-15; Thermo Fisher), supplemented with 10% FBS, 1% P-S, 2% tryptose phosphate broth, and 1% nonessential amino acids (NEAA) at 28°C.

Viruses were generated from a CHIKV infectious clone (CHIKV La Réunion strain, 06-049; GenBank accession number AM258994) (48). An AvrII site (CCTAGG) was engineered into the 06-049 clone in position 7612 to facilitate insertion/cloning back of the *de novo* synthetic constructs Stop and SuperStop (Eurogentec) by ligation with T4 DNA ligase. All newly generated plasmids were Sanger sequenced in full (GATC Biotech). A detailed list of all introduced changes is provided in Table S1 in the supplemental material. Plasmids were transformed into XL-10 Ultracompetent bacteria, and minipreps were performed with a NucleoSpin plasmid kit (Macherey-Nagel). Plasmid DNA was linearized with NotI enzyme (New England Biolabs) and purified with a PCR purification kit (Macherey-Nagel) prior to *in vitro* transcription (SP6 mMESSAGE mMACHINE kit; Invitrogen). RNA was subsequently purified by phenol-chloroform extraction and ethanol precipitation. Ten micrograms of transcript was electroporated (GenePulser XCell electroporator; Bio-Rad) in BHK-21 cells, and after 48 h supernatant containing infectious virus was collected and passaged once to generate working infectious virus stocks that were kept at −80°C until use. Virus stocks were Sanger sequenced to verify genetic stability of the introduced mutations.

Viruses expressing nano-luciferase (NLuc) (Promega, Madison, WI) were generated by introduction of the NLuc gene in each infectious clone between positions 5206 and 5718. NLuc was PCR amplified using primers 5′-AGATTTCGTTGGGGACTGGC-3′ (forward) and 5′-CGACAGGTACGGTGCTCATT-3′ (reverse). PCR product was purified using a Macherey-Nagel PCR purification kit. Using the NEBuilder HiFi DNA Assembly Master Mix (New England Biolabs) at a molar ratio between the vector and insert of 1:1, the PCR product was introduced into each infectious clone in nsp3. Plasmids were transformed into NEB Turbo competent cells (New England Biolabs) and purified with a ZymoPURE plasmid Midiprep kit. Once the correct plasmid sequence was confirmed by Sanger sequencing, 10 μg of plasmid was linearized with NotI (New England Biolabs), purified using a Macherey-Nagel PCR purification kit, *in vitro* transcribed using SP6 mMESSAGE mMACHINE kit (Invitrogen), and purified by phenol-chloroform extraction and ethanol precipitation. Two micrograms of transcript was used for transfection with Xfect transfection reagent in BHK-21 cells. After 48 h, virus was recovered, Sanger sequenced, aliquoted, and stored at −80°C.

### Virus titrations.

Vero cells were seeded into 24-well plates at 3 × 10^6^ cells per plate on the day prior to titration. Virus supernatants were serially diluted (10-fold) in serum-free DMEM, and 100 μl of each was transferred to confluent cells for 1 h at 37°C. After incubation, an overlay of DMEM, 2% FBS, and 0.8% (wt/vol) agarose (Invitrogen) was added. Three days after infection, cells were fixed with 4% formalin and stained with 0.2% crystal violet, and PFU were counted.

### Replication kinetics and virus infections.

Growth kinetics of viruses were determined in BHK-21, Vero, 293T, C6/36, Aag2, and U4.4 cells. Cells were seeded on the day before infection to 80 to 90% confluence for mammalian cells and at 60 to 70% confluence for insect cells. Viruses were diluted in serum-free medium to a multiplicity of infection (MOI) of 0.1, and cells were infected for 1 h at 37°C or 28°C. Following incubation, virus was removed, cells were washed with PBS, and medium containing 3% FBS was added. Supernatants were collected at different time points, and virus titers were determined by plaque assay.

### RNA extraction and quantification.

RNA was extracted from viral supernatants with TRIzol reagent (Invitrogen), and genome copy number was determined by a TaqMan RNA-to-*C_T_* (where *C_T_* is threshold cycle) one-step RT-PCR kit (Applied Biosystems). Each reaction was performed in the presence of 5 μl of RNA, 100 μM forward primer (5′-TCACTCCCTGCTGGACTTGATAGA-3′) and reverse primer (5′-GGGGTACTGTTCATCTGCTCTAAA-3′), and 25 pmol of probe (5′-[6-FAM]-AGGTACGCGCTTCAAGTTCGGCG-3′, where FAM is carboxyfluorescein) in an ABI 7000 machine. The reaction was performed at 50°C for 30 min, followed by a 10-min incubation at 95°C for the reverse transcription step and then by 40 cycles at 95°C for 15 s with extension at 60°C for 1 min. A standard curve was included in each run derived from *in vitro*-transcribed CHIKV RNA.

For quantification of viremia in mice, sera were separated from whole blood using BD Microtainer blood collection tubes and a quick centrifugation step. Then, sera were directly heated at 60°C for 5 min to release viral RNA and inactivate infectious virus (49). Genome copy number was determined using a Luna Universal One-Step RT-qPCR kit (New England Biolabs). Each reaction mixture contained 2 μl of a 1:5 dilution of RNA, 100 μM forward primer (5′-TCACTCCCTGCTGGACTTGATAGA-3′) and reverse primer (5′-GGGGTACTGTTCATCTGCTCTAAA-3′), 1.5 M trehalose, 5 M betaine, and 20 mg/ml bovine serum albumin (BSA) in an ABI 7000 machine. The reaction was performed at 55°C for 20 min, followed by a 5-min incubation at 95°C for the reverse transcription step and then by 40 cycles at 95°C for 10 s and extension at 60°C for 1 min. A standard curve was included in each run derived from *in vitro*-transcribed CHIKV RNA.

### Antiviral treatments.

Ribavirin (1-[(2*R*,3*R*,4*S*,5*R*)-3,4-dihydroxy-5-(hydroxy-methyl)oxolan-2-yl]-1*H*-1,2,4-triazole-3-carboxamide), azacytidine [4-amino-1-*b*-d-ribofuranosyl-1,3,5-tria-zin-2(1*H*)-one] (AZC), brequinar [6-fluoro-2-(2'-fluoro-1,1'-biphenyl-4-yl)-3-methyl-4-quinolinecarboxylic acid], and mycophenolic acid [(E)-6-(4-hydroxy-6-methoxy-7-methyl-3-oxo-1H-2-benzofuran-5-yl)-4-methylhex-4-enoic acid] (MPA) (Sigma Alderich) were diluted in dimethyl sulfoxide (DMSO) to a working stock solution.

Vero cells were pretreated for 2 h with each drug (200 μM and 400 μM ribavirin, 50 μM and 100 μM azacytidine, 0.5 μM and 2 μM brequinar, and 20 μM and 50 μM mycophenolic acid), followed by infection with viruses for 1 h at 37°C at an MOI of 0.1 PFU/cell, after which virus was removed, cells were washed twice, and medium (DMEM, 3% FBS, 1% P-S) containing antiviral drugs was replenished. Infected cells were incubated for 48 h at 37°C. Virus titers were determined by plaque assay.

### Plaque size measurement.

Subconfluent monolayers of Vero cells (10^7^) in 10-cm petri dishes were infected as described previously with 100 PFU of viral supernatants obtained from the endpoint of the antiviral treatment experiment. After incubation for 1 h at 37°C, virus was removed, and cells were washed twice with PBS and covered with an overlay of DMEM containing 3% FBS and 0.8% agarose. At 66 h after infection, 4% formalin was added to fix the cells, agarose plugs were removed, and cells were stained with crystal violet. Plaque size was quantified using ImageJ software (http://rsbweb.nih.gov/ij).

### Mosquito infections.

Aedes albopictus Providence (ALPROV) mosquitoes (F8 generation) from La Reunion were used for standard mosquito infections. Each virus was diluted to 3 × 10^6^ PFU/ml and mixed with 2 ml of previously washed rabbit blood supplemented with 5 mM ATP. Female mosquitoes were allowed to feed on artificial blood meals at 37°C for 30 min, after which engorged mosquitoes were selected for further incubation at 28°C with 10% sucrose *ad libitum*. Ten days after the blood meal, mosquitoes were killed, and full bodies were collected into 2-ml round-bottom tubes containing 200 μl of PBS and steel balls and homogenized in a TissueLyser II (Qiagen) at 30 shakes/s for 2 min. Viral titer was determined by plaque assay.

### Mouse husbandry and ethics.

Adult mice were housed in Institut Pasteur’s animal facility in biosafety level 3 (BSL-3) isolators and handled in compliance with the Animal Committee regulations and guidelines of Institut Pasteur Paris France under the 2010/63 European Union Council directive. Animal protocols were approved by the Ethics Committee on Animal Experimentation (CETEA) under dossier number 2013-0012/dap 160115/CHCST 10.620. Water and food were supplied *ad libitum*. Mice were monitored daily. End points were defined, and mice were humanly euthanized when they were reached. For mice handled in New York University (NYU), New York, USA, studies were performed according to approved protocols by the NYU School of Medicine Institutional Animal Care and Use Committee (IACUC) under dossier number IA16-01783.

### Mouse infections.

Five-week-old C57BL/6 mice (Charles River) were given a ketamine (10 mg/ml)-xylazine (1 mg/ml) mix by intraperitoneal (i.p.) injection. Once mice were fully anesthetized, 10^4^ PFU of wild-type or mutant viruses in 50 μl of DMEM was administered via footpad injection (*n* = 5 per virus). At 1 and 2 days after infection, mice were anesthetized in the same fashion and bled from the facial vein. At 2 days postinfection, footpad swelling was measured with a digital caliper as the height at the midpoint of the hind feet, and immediately afterwards mice were euthanized by cervical dislocation. Footpad and target organs (spleen and liver) were harvested. Half of the organ/tissue was collected in Eppendorf tubes containing 500 μl of RNA Shield (Zymo Research) to preserve RNA, and the other half was kept in Precellys tubes for hard or soft tissues that contained ceramic beads and 500 μl of PBS. Samples were either stored at −80°C or immediately homogenized in a Precellys 24 homogenizer (Bertin Technologies) at 5,000 rpm for 2 cycles of 20 s (spleen and liver) or at 6,800 rpm for 2 cycles of 30 s (footpad). Homogenates were cleared for 5 min at 5,000 × *g*, and viral supernatant was used for direct titration through plaque assay or used for an amplification cycle in C6/36 cells prior to titration in Vero cells by 50% tissue culture infective dose (TCID_50_), as described before (28). Briefly, 10-fold dilutions of virus supernatant were used to infect in triplicate 2 × 10^4^ C6/36 cells or 1 × 10^4^ BHK-21 cells in 96-well plates. Plates were incubated for 3 days at 28°C or 37°C, after which, 25 μl of cell supernatants was used to infect fresh Vero cells (1 ×10^4^ cells/well in a 96-well plate) and incubated at 37°C for 4 days. Cells were fixed and stained with crystal violet, and viral titer was determined by TCID_50_.

Infections in neonates (6 days old) were performed by subcutaneous injection of 200 PFU/ml of each virus in the backs of the mice (*n* = 3 for wild-type virus, *n* = 4 for Stop virus, and *n* = 5 for SuperStop virus). After 6 days, mice were euthanized by decapitation, and target tissues were collected and homogenized as described previously. Viral load was determined by direct plaque assay, and genome copy numbers were determined by RT-qPCR.

### *In vivo* imaging.

Six-week-old C57BL/6 albino mice were anesthetized and injected in the left footpad with 5 × 10^4^ PFU of either wild-type virus expressing nano-luciferase (NLuc) or mutant viruses expressing NLuc or PBS (*n* = 5 per condition). At days 1, 4, 6, 10, 14, and 18 after infection, mice were anesthetized and administered a 1/10 dilution of the NLuc substrate furimazine (stock at 2 mg/ml) via i.p. injection. Ten minutes later, mice were imaged on their backs using a Tecan IVIS Spectrum imaging device. Exposure time was determined automatically, with 10 s for images taken at days 1 and 4 postinfection and 60 s for images taken from day 6 onwards. Quantification of NLuc was performed using Living Image software (IVIS Imaging software) by defining specific areas of interest for infected and negative-control mice. NLuc measures were normalized by the value of the negative control for each group and time point.

### Protection studies.

Five-week-old C57BL/6 mice were injected in the footpad as described previously with 10^4^ PFU in 50 μl of wild-type or mutant viruses or PBS (*n* = 5 per condition). Mice were monitored daily. Four weeks after infection, mice were anesthetized as described above and bled for up to 600 μl from a facial vein into a BD Microtainer tube. Immediately, mice were euthanized by cervical dislocation. Serum containing neutralizing antibodies was separated from whole blood by centrifugation for 5 min at 5,000 rpm and 4°C. Supernatants were inactivated for virus and complement system for 1 h at 56°C. Sera from each group were pooled to the volume of the sample with less quantity. An aliquot was saved for performing *in vitro* neutralization assays. On the day prior to challenge, 100 μl of pooled sera was passively transferred to naive mice by i.p. injection (same group conditions, *n* = 5). In parallel, 0.1 mg of type I IFN-α/β receptor antibody (BD Biosciences) was administered to mice by i.p. injection to increase their susceptibility to CHIKV infection. The following day, mice were anesthetized and challenged with 10^4^ PFU in 50 μl of wild-type CHIKV via footpad injection. A control group of mice received only the type I IFN-α/β receptor antibody and were not challenged. Mice were monitored for survival and weight change, footpad swelling was measured from day 3 onwards, and viremia was determined at 1, 3, 4, 5, 7, and 9 days postinfection.

### Neutralization assays.

To quantify neutralizing antibodies obtained in the sera, we used a luciferase-based neutralization assay that requires the use of a nano-luciferase-expressing CHIKV (NLuc-CHIKV) stock. NLuc-CHIKV was diluted to a working concentration of 10^5^ PFU/ml in DMEM and 2% FBS, and 195 μl was incubated with 5 μl of each serum sample (previously inactivated for 30 min at 56°C) at 4°C overnight. Fifty microliters of this mix was added to a 96-well plate (black), previously seeded with BHK-21 cells, and incubated for 1 h at 37°C. After infection, cells were washed with PBS, and 2% FBS-DMEM was replenished to each well. Cells were incubated for 6 h and lysed, and 50 μl of Nano-Glo reagent was added to each well. The plate was read in a luminometer, and neutralizing antibody titers were expressed as fold reduction in luminescence relative to that with mock treatment.

### Transmission studies.

Transmission experiments were conducted at New York University, New York, USA, under BSL-3 conditions. Aedes aegypti mosquitoes (1 colony, F15 generation, collected in Poza Rica, Mexico, in 2016) (50) were given an artificial blood meal with 10^6^ PFU/ml (as described before), and after feeding, engorged mosquitoes were selected and incubated at 28°C. Fourteen days after blood meal, mosquitoes were food deprived for 12 h. Four-day-old neonatal mice were immobilized over a mesh covering the cup containing the infected mosquitoes to allow feeding for 40 min. Afterwards, mice were returned to their cages, and mosquitoes were killed and homogenized, and viral titers were determined by plaque assay. Mice were monitored daily for disease until 6 days after transmission, when they were sacrificed, and target organs (brain, leg muscle) were collected and homogenized as described previously. Viral titers were determined by plaque assay, and RNA levels were determined by qRT-PCR.

### Next-generation sequencing.

In order to quantify the number of stop codons introduced in the viral population of infected mosquitoes and mice, Illumina amplicon sequencing was performed. RNA was extracted using TRIzol reagent (Invitrogen), and cDNA was generated with a Maxima H Minus First Strand cDNA Synthesis kit (ThermoFisher) with random hexamer primers. Viral DNA was amplified with Platinum SuperFi DNA polymerase (Invitrogen) in two fragments of 2,765 bp and 2,638 bp that cover the full capsid and envelope regions, where our changes were introduced. Primers used were the following: the pair 5′-CACTACAGGAAGTACCAATGG-3′ (forward) and 5′-CAGCATTACGCGGGACCAGAG-3′ (reverse) and the pair 5′-GAAGCGACAGACGGGACG-3′ (forward) and 5′-CTATTCAGGGGTTGCGTAG-3′ (reverse). The reaction was performed at 98°C for 30 s, followed by 35 cycles at 98°C for 10 s, 60°C of annealing for 10 s, and extension at 72°C for 90 s. PCR products were purified with a Macherey-Nagel PCR purification kit and quantified with Quant-it Picogreen (ThermoFisher) solution in a Tecan machine. DNA was then fragmented (Covaris), ligated to adapters (NEBNext Ultra DNA; New England Biolabs), sequenced in an Illumina NextSeq 500 system, and analyzed with in-house scripts.

### Determination of stop codon frequencies.

Sequenced reads for each sample were trimmed using Trimmomatic (51) in order to remove low-quality bases and reads shorter than 36 nucleotides and aligned to their respective reference genomes using Burrows-Wheeler Aligner (BWA) (52). Codon frequencies were estimated using quality scores in the aligned data as described in Moratorio et al. (18). In brief, the observed codon frequencies were modelled by a multinomial model with noisy observations. Maximum likelihood estimation was used to infer the per-site codon frequencies for each sample based on this model. The computed frequencies were estimated using all the reads overlapping that position, ignoring bases with a Phred score of ≤30. Finally, per-sample stop codon frequencies were computed by summing the per-site stop codon frequencies over all modified codon sites. Bar plots were used to visualize and compare the frequency estimates for wild-type, Stop, and SuperStop samples.

### Statistical analyses.

Statistics were performed in GraphPad Prism software. *P* values of >0.05 were considered nonsignificant (ns). No samples or infected animals were excluded from analysis. For animal studies, mice were randomly allocated to different cages prior to experiments. The investigator was blinded to group allocation when virus was titrated from harvested tissues.

## Supplementary Material

Supplemental file 1
